# How can we make therapy better for autistic adults? Autistic adults’ ratings of helpfulness of adaptations to therapy

**DOI:** 10.1177/13623613251313569

**Published:** 2025-01-22

**Authors:** Jessica Paynter, Kristyn Sommer, Amanda Cook

**Affiliations:** Griffith University, Australia

**Keywords:** accommodations, adaptations, autism, mental health, therapy

## Abstract

Autistic people are at elevated risk of mental health conditions and experience significant barriers to effective support. While adaptations to therapy for autistic people have been proposed by clinicians, there is limited research on how helpful autistic people themselves rate these. We aimed to address this gap. Participants were 130 autistic adults aged 18 to 64 years (85.4% female) who completed an online survey of the helpfulness of 55 therapy adaptations. Overall, we found positive ratings of helpfulness for approximately half of the adaptations. However, significant variability was found at an individual level with most adaptations rated from not helpful through to extremely helpful. Neurodiversity-affirming adaptations were rated highest overall. Participants shared additional adaptations including general good practice, financial, modality, neurodiversity-affirming practices, practical, sensory/environmental, structure, and therapy style/techniques. Findings highlight similarities and differences between clinician and autistic people’s perspectives, underscoring the need to include autistic people. Limitations of the restricted sample and generalizability are acknowledged. Future research including more diverse samples would be of value to expand on this research. The need to understand general preferences of autistic people and to individualize to the specific client is underscored by findings to begin bridging the mental health support gap for autistic people.

**Lay Abstract:**

Autistic people experience more mental health conditions like depression or anxiety than non-autistic people. They are also more likely to experience difficulties in accessing mental health supports Clinicians have published suggestions on how to improve therapy for autistic people. However, whether these ways to adapt (i.e. adaptations) therapy for autistic people are seen as helpful by autistic people themselves has not been investigated. We recruited 130 autistic adults to complete an online survey. They rated 55 adaptations to therapy from “Not at all helpful” to “Extremely helpful.” We also asked for ideas of additional adaptations. Adaptations classified as neurodiversity affirming (e.g. having a therapist that embraces differences in brains and provides support to affirm neurodivergent identity) were rated highest. Approximately half of adaptations were rated positively at a group level. However, for almost every adaptation at least one person rated it as not at all helpful and at least one rated it as extremely helpful. Additional adaptations were around general good practice, financial cost, neurodiversity-affirming practices, practical, sensory/environmental, structure, and therapy style/techniques. Our findings add how helpful autistic people themselves rate adaptations to therapy and similarities and differences to clinicians. This is important to consider how these perspectives can differ. Findings also identify additional suggestions that clinicians could use in their practice and ideas for future research. Findings can help autistic adults in advocating for adaptations to therapy that address their needs by providing a list of possible adaptations. Furthermore, findings may help clinicians to better support their autistic clients.

Autistic^1^ people are at elevated risk of mental health conditions (Lever & Geurts, 2016), negatively impacting quality of life (Lawson et al., 2020) and contributing to premature mortality (Hirvikoski et al., 2016). Furthermore, autistic people face significant barriers to accessing effective supports for their mental health (Brede et al., 2022; Camm-Crosbie et al., 2019). Barriers include services being based around neurotypical norms, clinicians’ lack of awareness, stereotyping, and resistance to adapting services to autistic people’s needs (Adams & Young, 2021; Brede et al., 2022). Autistic adults additionally raise challenges from differing communication styles, expression/identification of emotions, thinking styles, and sensory processing (Brede et al., 2022). Furthermore, autism-specific presentations (e.g. depression, see review, Hinze et al., 2024) and mechanisms have been identified for mental health conditions (e.g. intolerance of uncertainty and anxiety, see review Jenkinson et al., 2020) indicating a potential need to adapt therapies for this population. Finally, clinicians report lower confidence working with autistic clients than other client groups (Gallant et al., 2022) further exacerbating challenges in accessing effective supports tailored to this group’s needs. Recent clinician research provides important insight into potential adaptations to therapy clinicians could make to improve therapy for autistic people (Cooper et al., 2018; Petty et al., 2021). There is however limited research into the implementation of specific adaptations including how helpful autistic people themselves feel proposed adaptations would be to them. We address this important gap to explore how useful recommended adaptations to therapy are rated by autistic adults who have seen a psychologist for therapy. Co-designed adaptations with autistic people are essential to addressing the needs and strengths of this population for more suitable, and effective, mental health support.

## Autism characteristics and mental health

Autistic people experience and interact with the world in ways that differ from the majority neurotypical norms in social and communication styles, engagement in passions and interests, and sensory processing. These differences may place autistic people at greater risk for mental health conditions. For example, approximately 50% of autistic individuals, relative to 5% of neurotypical individuals, report difficulties with identifying and expressing emotions (i.e. alexithymia; Kinnaird et al., 2019). Alexithymia is associated with greater anxiety in non-autistic individuals (Berthoz et al., 1999) and autistic adolescents (Milosavljevic et al., 2016). In addition, differences in emotion regulation, intolerance of uncertainty, and sensory processing have been implicated in vulnerabilities to mood and anxiety conditions for autistic people (Conner et al., 2022; Glod et al., 2015; Jenkinson et al., 2020). Furthermore, mental health conditions may be expressed in differing ways by autistic people to neurotypical expressions, and/or may overlap with, or be overshadowed by, autism characteristics (Adams et al., 2018; Rosen et al., 2018). Thus, the interaction of autism-specific differences in a majority neurotypical society may lead autistic people to being more vulnerable to mental health challenges and for individuals to experience greater barriers to receiving effective support.

## Autism and mental health experiences: autistic perspectives

Autistic people report facing significant barriers to accessing effective mental health support (Brede et al., 2022; Camm-Crosbie et al., 2019). Brede et al. (2022) conducted a systematic review and meta-synthesis of qualitative studies that investigated autistic adults’ experiences of accessing and receiving support for mental health difficulties. Included studies were with autistic adults (*n* = 24), professionals/clinicians (*n* *=* 13), and family members (*n* = 10), with eight studies including overlapping groups. They identified 38 individual studies, mostly from the United Kingdom (26/38), or the United States (7/38), with no studies from Australia or New Zealand. Findings clustered into three themes: lonely, difficult service experience; complexity needs flexibility; and collaboration and empowerment. The lonely, difficult service experience included subthemes of barriers at each step (e.g. services based on neurotypical norms) and negative consequences of accessing services (e.g. harm from supports). Complexity needs flexibility consisted of subthemes of the impact of being autistic on treatment (e.g. autism/mental health interaction) and the need for a comprehensive and flexible approach (e.g. adjusting timings/expectations). Collaboration and empowerment included a focus on relationships therapeutically including listening to autistic perspectives and enablement of independence, self-advocacy, and self-care. Of note, inspection of included papers indicated that none looked specifically at ways mental health supports in general could be tailored to autistic people, and predominantly explored experiences broadly, subsequently identifying challenges only, rather than potential solutions. Similarly, a recent quantitative review of barriers and facilitators to accessing psychological treatment for mental health for autistic people found facilitators were rarely raised in previous research (Adams & Young, 2021). Emerging research has explored Autistic adults’ perspectives on acceptability of adapted therapies as part of research studies. For example, adapted therapy for depression in autistic adults, which found participants valued therapists showing a good understanding of autism and using explanations given in a level of detail they could understand (Horwood et al., 2021). However, a significant gap in the literature of autistic adults’ perspectives on ways to improve therapy in community-based support exists.

## Adaptations to therapy: clinician perspectives

Given the dearth of research into facilitators of effective mental health care in the community from autistic perspectives, a starting point may be to understand recommended adaptations to therapy from clinician perspectives. Cooper et al. (2018) investigated adaptations to cognitive behavior therapy (CBT) in the United Kingdom for autistic adults using a survey design to evaluate the frequency of use of 12 potential adaptations to CBT with 50 therapists. They found most participants reported making adaptations to CBT for autistic clients with the most common: using more behavioral strategies to introduce change (74%), using plain English more often (70%), using a more structured and concrete approach to therapy (68%), providing more written or visual information (60%), and including interests/hobbies in therapy (58%). While this study provides some potential suggestions, the use of a limited list of only 12 possibilities and closed-ended methodology, and focus on one specific therapy modality, limits the generalization and depth of understanding that may be drawn from this study.

Petty and colleagues’ (Petty et al., 2021, 2023) investigations using interview-based open-ended exploration of potential adaptations to therapy provide further insight into clinicians’ perspectives. Petty et al. (2021) developed a list of recommendations for adapting services for autistic clients using a free listing approach via interviews where 15 staff from a multidisciplinary specialist autism service in the United Kingdom were asked to outline ways they adapt their practice for autistic clients. Adaptations identified included consideration of the sensory environment (e.g. client’s sensory needs), adapting communication (e.g. verbal/written communication/materials), and minimizing client uncertainty (e.g. providing service information in advance). More recently, Petty et al. (2023) conducted semi-structured interviews with seven clinicians from a specialist assessment and therapy service in the United Kingdom. They described adaptations for autistic adults. Key themes included consideration of autism characteristics (e.g. intolerance of uncertainty, sensory sensitivities), clinician adaptations (e.g. using visuals), service adaptations (e.g. providing information in advance), and service improvements (e.g. staff autism training). These clinician suggestions provide insight into perceptions of those delivering services; however, there is a need to evaluate whether such recommendations are experienced as helpful by autistic people themselves.

## Present study

Previous research has established the high prevalence of mental health conditions experienced by autistic people and the substantial barriers autistic people face in accessing mental health support. Emerging research documents potential adaptations to therapy to improve outcomes and experiences for autistic people. However, this is predominantly from clinicians’ perspectives and has not been validated by autistic people themselves. This is essential to ensure adaptations meet the needs of autistic people and to identify further possibilities. Furthermore, research has been predominantly in the United Kingdom within specific contexts; the mental health context, funding models, and clinician training may differ in the United Kingdom from other parts of the world. There is a need for specific research in Australia and New Zealand, which share geographical proximity and similar systems including a mixture of privately and publicly funded mental health services. We include autistic people with both formal and self-identified diagnoses to reflect clinical reality and the privilege and cost of formal diagnostic assessment with exploration of potential group differences incorporated into the study design, consistent with previous service delivery research (e.g. Hampton et al., 2023). Our aim was to understand autistic people’s perspectives on the helpfulness of adaptations raised by clinicians (Cooper et al., 2018; Petty et al., 2021, 2023) with a key research question of how helpful each adaptation is rated by autistic people. We also explored as a secondary aim whether we could group adaptations into categories and what categories of adaptations were rated as most helpful. In these categories, we explored whether helpfulness differs by self-identified versus formal diagnosis. Finally, we explored what other adaptations were raised by participants. Given the paucity of research in this area no specific hypotheses were made.

## Method

### Context

This research was conducted with participants across Australia and New Zealand. Across these countries, mental health care is provided through a mixture of private (e.g. private psychologists) and public health services (e.g. hospital mental health services). Of note mental health is excluded from Australia’s National Disability Insurance Scheme (National Disability Insurance Agency, 2024). Mental health is included under their medical system which includes limited items for mental health. These items provide a rebate for services with a general practitioner (i.e. primary care physician) or specialist professional (e.g. psychiatrist or psychologist) in public or private settings. Private practitioners can charge higher fees than these item rebates that result in gap fees for clients which can be over $100 per session (Rosenberg et al., 2022). The Royal Australian and New Zealand College of Psychiatrists has highlighted the significant unmet needs and challenges for autistic people in accessing mental health supports across these countries (The Royal Australian & New Zealand College of Psychiatrists, 2022). They also raise the challenge of a lack of consistent data collection for autistic people specifically across Australia and New Zealand meaning it is difficult to ascertain current levels of mental health care and engagement. However, the unmet needs have been acknowledged by Australia’s government and a Roadmap for Health and Mental Health for Autistic Australians is in development (Australian Government: Department of Health and Aged Care, 2024).

### Design

An online survey design was used. The project was pre-registered on 29 March 2023 on the Open Science Framework (OSF; https://osf.io/8hd54). The project had ethical approval from the Griffith University Human Ethics Committee (Ref No: 2023/183), and all participants provided informed consent after viewing an information sheet and completing a consent form to access the online survey.

### Participants

Participation was voluntary and included an optional prize draw (one of two $50 gift vouchers; drawn 1 September 2023), with participants provided a summary of therapy adaptations included in the study at survey completion (available via OSF page, https://osf.io/hq3x5) and sent a summary of findings at project completion.

Inclusion criteria were being an autistic adult (18 years or older) including formally diagnosed or self-identifying, having seen a psychologist for therapy, living in Australia or New Zealand, self-selected ability to read and write in English, and completion of the key measures (adaptations in therapy and demographics) in the online survey. All participants completed the Autism Quotient-10 (AQ-10, Booth et al., 2013) for descriptive purposes. Participants in the self-identifying group showed a mean score above the AQ-10 cut-off of six (*M* = 6.66, *SD* = 1.84) and did not show significantly different scores to participants with a formal diagnosis (*M* = 7.31, *SD* = 1.97), *t*(125) = 1.903, *p* = 0.06, *d* = 0.34.

The survey was completed by 223 people with 137 completing the key measures. Seven participants were excluded due to living outside of Australia or New Zealand (*n* = 6) or not reporting their country (*n* = 1). The final sample included 130 participants. Participants were predominantly from Australia, with most participants female and aged 18 to 44, having post-secondary qualifications, with similar proportions of participants reporting formal (53.8%) and self-identified diagnoses (46.2%) (see Table 1).

**Table 1.** Participant demographics.

Variable		Number (%)	
Autism diagnosis			
Formal		70 (53.8%)	
Autism Spectrum Disorder (DSM-5)		60 (85.7%)	
Autistic Disorder (DSM-IV)		0	
Asperger’s Disorder/Syndrome (DSM-IV)		8 (11.4%)	
Other		2 (2.9%)	
Self-identified		60 (46.2%)	
Gender			
Male		5 (4%)	
Female		111 (85.4%)	
Non-binary		9 (6.9%)	
Another gender		1 (.8%)	
Age bracket			
18–24		18 (13.8%)	
25–34		71 (54.6%)	
35–44		36 (27.7%)	
45–54		4 (3.1%)	
55–64		1 (.8%)	
Highest level of education			
Junior certificate/grade 10 equivalent		1 (.8%)	
Senior certificate/grade 12 equivalent		10 (7.7%)	
Vocational certificate or diploma		34 (26.2%)	
Bachelor’s degree		46 (35.4%)	
Postgraduate degree		36 (27.7%)	
Other		3 (2.3%)	
Work status			
Full-time		45 (34.6%)	
Part-time		30 (23.1%)	
Casual		10 (7.7%)	
Studying		14 (10.8%)	
Home duties/caring		15 (11.5%)	
Unemployed/looking for work		5 (3.8%)	
Disability support/pension		5 (3.8%)	
Other		3 (2.3%)	
Prefer not to answer		3 (2.3%)	
Country			
Australia		123 (94.6%)	
New Zealand		7 (5.4%)	
Ancestry			
Australian		107	
Australian Aboriginal		2	
New Zealander		8	
Māori		1	
British		35	
Irish		24	
Scottish		12	
Chinese		2	
Italian		3	
Maltese		3	
Dutch		6	
Other		9	
Co-occurring diagnoses	Suspected	Self-identify	Formally diagnosed
Attention Deficit Hyperactivity Disorder	23 (18.7%)	18 (14.6%)	63 (51.2%)
Anxiety Disorder	9 (7.1%)	10 (7.9%)	89 (70.6%)
Depression	8 (6.6%)	5 (4.1%)	72 (55.4%)
Eating Disorder	18 (16.2%)	9 (8.1%)	13 (11.7%)
Gender Dysphoria	4 (3.7%)	3 (2.8%)	2 (1.5%)
Learning Disorder	10 (8.9%)	7 (5.4%)	13 (10%)
Obsessive-Compulsive Disorder	18 (16.1%)	4 (3.6%)	15 (13.4%)
Substance Use Disorder	4 (3.1%)	3 (2.8%)	2 (1.8%)

### Measures

#### Demographics

Questions covered autism diagnosis, co-occurring conditions, gender, age bracket, education, work status, country, and ancestry. Participants were also asked about their most recent therapy experiences including perceived helpfulness (5-point Likert-type scale, not at all helpful to extremely helpful), years of therapy and what they had sought mental health support for to contextualize responses. Measure available via OSF (https://osf.io/t6xpa).

#### Autism characteristics

The AQ-10 (Allison et al., 2012) asks participants to rate their level of agreement with 10 statements with items scored dichotomously with a clinical cut-off of six or higher. The AQ-10 shows good sensitivity and specificity in discriminating autistic from non-autistic individuals (Booth et al., 2013). The AQ-10 was used to describe the number of autism characteristics endorsed for descriptive purposes.

#### Adaptations to therapy scale

This scale was a 55-item Likert-type scale (1, *not at all helpful* to 5, *extremely helpful*) along with an open-ended question to overview additional adaptations participants had found helpful in previous therapy. This scale was developed for the present study drawing from previous research (Cooper et al., 2018; Petty et al., 2021, 2023), with additional adaptations drawn from lived experience of the second author and clinical experience of the first author, and feedback from two autistic adults who pilot-tested the measure. The initial survey was drafted by the first author, reviewed for appropriateness and wording by the second author and refined. Then, it was pilot tested by two autistic adults. Feedback included providing more specific examples (around recognizing emotions, thoughts, and goals items), rephrasing item wording (remove ‘I’ where not needed), and to add one item (low sensory waiting room) which were incorporated in the final survey. It was subsequently entered into the Redcap survey platform and a final test completed by an autistic reviewer before finalizing for distribution. The full scale is available via OSF (https://osf.io/t6xpa).

### Procedure

Participants were recruited via social media, autism services, and therapists (waiting room flyers). Data were collected between 28 March 2023 and 23 May 2023. Target participant numbers (100) were reached after approximately 1 week.

### Data analysis and screening

Deidentified data are available via OSF (https://osf.io/8ekch). No outliers or patterned responses were identified, and missing data were minimal (did not report gender, *n* = 4 (3.1%): did not complete AQ-10, *n* = 3 (2.3%)). There was no missing data for therapy adaptations items. For individual items, comparison of participants with a formal diagnosis and a self-identified diagnosis were explored with no clear patterns between groups and were thus combined for analysis. Data were inspected for the full sample versus females only given the small number of males with no substantive differences observed thus the full sample were retained across analyses.

Descriptive statistics were conducted to overview demographics and contextualize participants’ therapy experiences. To address how helpful each individual adaptation was rated, descriptive statistics were conducted including calculating mean, standard deviation, range, and percentage of respondents rating each item as helpful (rating of 4 or 5 with 5 indicating extremely helpful). To address what categories of adaptations are rated as most helpful, individual adaptations were classified by authors one and two into categories, based on clinical experience and categories from previous research items were drawn from (Cooper et al., 2018; Petty et al., 2021, 2023). These logically derived subcategories demonstrated face and content validity as evaluated by the authors, with internal consistency evaluated to assess reliability, a variation from the pre-registered protocol (exploratory factor analysis which did not yield cohesive categories deemed meaningful for interpretation by authors one and two). Categories included: Communication, Information, Modality, Neurodiversity Affirming, Session Duration, Sensory, Techniques, and Visuals Adaptations (see Table 3). Session Duration, Modality and Visuals had poor internal consistency thus were excluded from further analysis. Remaining subscales had acceptable or good internal consistency. Subscale totals were computed by averaging items in subscales.

A within-participants analysis of variance was conducted to compare category ratings. Data screening showed outliers; however, inspection suggested genuine, non-patterned responses, with no substantive impact on analysis or assumptions and were consequently retained. No other major violations of assumptions were detected, outside of sphericity (Mauchly’s test of sphericity, χ^2^(9) = 29.40, *p* < 0.001) with a Greenhouse-Geisser correction consequently applied (ε = 0.902) for analysis. To address what other adaptations may be helpful, authors one and two independently read and identified codes (i.e. discrete ideas) in open-ended responses to identify additional unique specific adaptations raised by participants. They had 100% concordance of identification of the unique codes and discussed naming of codes and combining codes together that addressed similar ideas (e.g. advice on who can give diagnosis with help finding therapists as being about support navigation). They then discussed to consensus the list of unique adaptations classified these into categories using directed content analysis (Hsieh & Shannon, 2005). This included developing definitions of existing categories and codes fitting these; and developing definitions of codes and groupings of codes that did not fit in existing categories. To evaluate reliability of coding, a blind coder then classified quotes into codes after being provided definitions of each code, and these definitions into categories. Coding quotes to codes showed very good (Altman, 1999) interrater reliability (κ = 0.96) with only two discrepancies resolved to consensus after clarifying definitions of communication and explicit consent. Coding definitions of codes to categories showed good reliability (κ = 0.64) based on Cohen’s Kappa (Altman, 1999). Discrepancies were discussed to consensus with coding clarified linking quotes to codes. Discrepancies were predominantly around differentiating communication (of therapist) from client; structure (defined as in-session) versus practical (clarified as around or outside of session) adaptations and selecting specific (sensory or communication) over the broader techniques category.

To address the final research question, *t*-tests were conducted to compare the helpfulness of adaptations ratings for the derived categories by formal versus diagnosis group. Alpha was set at 0.05 across analyses.

### Community involvement

We used a co-design approach with an emphasis on transparency, reproducibility, and inclusive practice, drawing from autistic lived experience and clinical (psychology) experience across the authorship team. Our project conception and focus on mental health was informed by mental health being a high priority area for autistic people in community priority setting studies (Roche et al., 2021). We aligned our approach with guidelines for inclusive practice (den Houting, 2021). This included co-design across our autistic and non-autistic authorship team from project initial development, selection and development of measures, data analysis, interpretation, and dissemination including manuscript preparation.

## Results

### Recent therapy experiences

Participants reported seeking psychological therapy between 2014 and 2023, most commonly for anxiety (*n* = 103, 79.2%), followed by depression (*n* = 74, 56.9%), and relationships (*n* = 42, 40%) (see Table 2). Approximately 54% of participants reported their most recent therapy was helpful or extremely helpful, 22% rated it as neutral, and 24% rated it as unhelpful or not at all helpful (*M* = 3.48, *SD* = 1.24, range = 1–5).

**Table 2.** Reasons for psychological therapy.

Reason	*n* (%)
Attention-deficit hyperactivity disorder	8 (6.2%)
Autism diagnosis	4 (3.1%)
Anxiety	103 (79.2%)
Depression	74 (56.9%)
Eating disorder	16 (12.3%)
Gender dysphoria	5 (3.8%)
Learning or studying	16 (12.3%)
Obsessive-compulsive disorder	3 (2.3%)
Post-traumatic stress disorder	9 (6.9%)
Relationships	52 (40%)
Substance abuse	3 (2.3%)
Trauma	10 (7.7%)
Work or vocational supports	21 (16.2%)
Other	28 (21.5%)

### Perceived helpfulness of individual adaptations

At an individual level most (51/55, 92.73%) adaptations showed the full range of scores (1–5) indicating at least one person felt an adaptation could be not helpful at all (1) or extremely helpful (5). The remaining four adaptations at least some participants rated as low in helpfulness (2) to extremely helpful. No individual adaptation was universally endorsed (i.e. only a 4 or 5) by all participants. Ratings for the helpfulness of each individual adaptation ranged from *M* = 2.1 (shorter sessions) at a group level, with 11.54% rating this as helpful to *M* = 4.78 (psychologist high level knowledge about autism) at a group level, with 96.15% of participants rating this as a four or higher indicating positive ratings of helpfulness (see Table 3).

**Table 3.** Descriptive statistics for each therapy adaptation by subscale (*N* = 130, ordered by highest to lowest % rating helpful).

Adaptation^a^	Internal consistency	Mean (SD)	Range	% Rating helpful (Item rated > 3)
**Communication**	Acceptable (0.722)	3.71 (0.67)		
Psychologist . . .				
Explained terminology in measures/questionnaires		4.33 (0.94)	1–5	85.38
Let you know that you are not expected to share eye contact		4.28 (1.12)	1–5	82.31
Provided support to complete questionnaires/measures		3.82 (1.22)	1–5	67.69
Used more closed questions to explore emotions		3.71 (1.18)	1–5	61.54
Provided response options (e.g., writing/drawing) instead of talking		3.55 (1.34)	1–5	55.38
Provided written materials		3.54 (1.18)	1–5	53.08
Used plain English instead of technical language/jargon		3.55 (1.19)	1–5	49.23
Avoided using metaphors/figurative language		3.42 (1.39)	1–5	48.46
Changed their body language/facial expressions/eye contact to your preferences		3.22 (1.28)	1–5	43.85
**Information**	Acceptable (0.786)	4.15 (0.65)		
The clinic provided information about . . .				
What to expect in therapy before starting therapy with a new clinician/clinic		4.48 (0.79)	2–5	89.23
Location and people on their website		4.34 (0.79)	2–5	86.15
Therapy process		4.34 (0.89)	1–5	86.15
Psychologist provided the questions for sessions in advance		4.15 (1.07)	1–5	80.77
Psychologist provided written information about supports available outside of sessions		4.28 (1.00)	1–5	79.23
The clinic provided information about location and people in advance		4.02 (1.16)	1–5	71.54
The clinic provided written and visual information about staff members in office/building		3.95 (1.09)	1–5	69.23
Psychologist gathered initial information through e-mail/letter/telephone		3.68 (1.29)	1–5	62.31
**Modality**	Poor (0.589)			
Online booking options		4.78 (0.64)	1–5	96.15
Therapy in individual sessions		4.56 (0.72)	1–5	92.31
Text booking option		4.50 (0.99)	1–5	86.92
E-mail appointment option		4.28 (1.03)	1–5	78.46
Therapy provided online via video conferencing		3.19 (1.44)	1–5	44.62
Psychologist involving a family member or partner in sessions		2.60 (1.30)	1–5	26.15
Therapy provided in groups with other autistic people		2.32 (1.13)	1–5	13.08
Therapy provided via telephone		2.14 (1.23)	1–5	13.08
**Neurodiversity affirming**	Good (0.810)	4.22 (0.60)		
Psychologist . . .				
High level of knowledge about autism		4.78 (0.53)	2–5	96.15
Used a neurodiversity-affirming approach		4.71 (0.60)	2–5	93.85
Used supports/therapies developed by/with autistic people		4.45 (0.88)	1–5	88.46
Used questionnaires/measures designed by/with autistic people		4.48 (0.86)	1–5	85.38
Psychologist . . . Accepted diagnosis/self-identification when you were seeking support for a different issue or goal		4.44 (0.94)	1–5	84.62
Worked with other health/ allied health professionals supporting your needs		4.16 (1.09)	1–5	77.69
Asked/used your preferred terminology to talk about autism		4.14 (1.25)	1–5	73.85
Asked if you would like to revisit or formalize your autism diagnosis/self-identification		4.02 (1.23)	1–5	70.00
Incorporated your hobbies/interests into therapy		3.82 (1.09)	1–5	68.46
Disclosed if they identify as autistic		3.77 (1.27)	1–5	63.85
Identified as autistic themselves		3.67 (1.23)	1–5	60.77
**Sensory**	Good (0.855)	4.05 (0.80)		
Psychologist provided . . .				
Options to adjust the lighting		4.22 (0.98)	1–5	80.00
Your psychologist providing sensory or twiddle items in sessions		4.29 (1.05)	1–5	83.08
Options to adjust the noise		4.16 (1.05)	1–5	76.15
The clinic provided a low sensory environment for therapy		4.12 (0.98)	1–5	74.62
The clinic provided a low sensory waiting room		4.04 (1.07)	1–5	71.54
The clinic provided a space to use after a session to relax or recharge if needed		3.83 (1.24)	1–5	66.92
Your psychologist provided the option to take breaks in sessions		3.66 (1.21)	1–5	61.54
Session duration	Poor (−0.551)			
Sessions were about an hour		3.78 (0.94)	1–5	62.31
Sessions were longer		3.35 (1.40)	1–5	49.23
Your psychologist changing pace in sessions		3.12 (1.05)	1–5	30.77
Sessions were shorter		2.10 (1.14)	1–5	11.54
**Techniques**	Acceptable (0.713)	3.87 (0.82)		
Your psychologist spent more time to get to know you before working on your goals		4.11 (1.02)	1–5	76.92
Your psychologist included techniques to identify and express . . .				
Goals and values in therapy		3.85 (1.10)	1–5	66.92
Your psychologist included techniques to successfully identify and express your thoughts in therapy		3.82 (1.16)	1–5	66.15
Emotions in therapy		3.68 (1.18)	1–5	62.31
**Visuals**	Poor (0.597)	3.65 (0.77)		
Visuals in therapy		3.94 (0.95)	1–5	73.08
Visual of time left in each session		3.56 (1.42)	1–5	60.00
Psychologist using . . . Visual/list of activities of session content		3.60 (1.11)	1–5	55.38
Structured and concrete approach to therapy		3.48 (1.15)	1–5	51.54

a Note adaptations have been abbreviated due to space restrictions, for the verbatim measure see: https://osf.io/t6xpa.

Of the 55 adaptations, 26 (47.27%) had a mean rating of 4 or 5 indicating they were perceived at a group level as helpful or extremely helpful. These were also rated as helpful (rating of 4 or 5) by between 71.54% (information on locations and people provided) and 96.15% (psychologist high level knowledge of autism and online bookings) of individual participants. The highest rated adaptations were the psychologist having a high level of knowledge of autism, being able to book appointments online, the psychologist using a neurodiversity-affirming approach (e.g. embraces differences in brains and provides supports to affirm neurodivergent identity), therapy being provided in individual sessions, and the clinic providing an option to text to book appointments, with each of these rated on average 4.5/5 (5 = extremely helpful) and rated four or five (helpful) by 86.92% (text appointment) or more of individual participants. A further 25/55 (45.45%) had a mean rating between 3 and 3.95 indicating they were rated as mid-range of helpfulness to helpful on average at a group level, for example, providing therapy online, longer sessions, and using visuals which all had mixed feedback (ratings 1–5, percentage rating as helpful [4–5] from 30.77% [changing pace] to 69.23% [information about staff]). Four possible adaptations had ratings below 3 (with 1 = not at all helpful, and 3 the mid-point of the scale), which were from lowest to highest, shorter sessions (<50 min), providing therapy on the telephone, group therapy with other autistic people, and involving a family member or partner in sessions. At an individual level between 11.54% (shorter sessions) and 26.15% (involving a family member or partner in sessions) rated these as helpful.

### Adaptation category helpfulness

There were statistically significant differences in ratings between categories of adaptations, *F* (3.609, 465.524) = 24.355, *p* < 0.001, partial η^2^ = 0.159. Highest to lowest categories based on means were neurodiversity affirming (*M* = 4.22, *SD* *=* 0.60), information (*M* = 4.15, *SD* *=* 0.64), sensory (*M* = 4.05, *SD* *=* 0.80), techniques (*M* = 3.87, *SD* *=* 0.82), and then communication (*M* = 3.71, *SD* *=* 0.67). Post hoc analysis with a Bonferroni adjustment showed that neurodiversity affirming adaptations were rated significantly higher than sensory, communication and technique adaptations, but equivalently to information (see Table 4). Communication adaptations were rated significantly lower than information, sensory and neurodiversity affirming, but equivalently to technique adaptations.

**Table 4.** Post hoc comparisons between categories of therapeutic adaptations (Bonferroni corrected).

Comparison	Mean difference (*SE*)	Significance
Neurodiversity Affirming versus Information	0.07 (0.05)	>0.999
Neurodiversity Affirming versus Sensory	0.175 (0.06)	0.032*
Neurodiversity Affirming versus Communication	0.51 (0.05)	<0.001***
Neurodiversity Affirming versus Techniques	0.35 (0.06)	<0.001***
Information versus Sensory	0.11 (0.07)	1.000
Information versus Communication	0.44 (0.05)	<0.001***
Information versus Techniques	0.29 (0.07)	<0.001***
Sensory versus Communication	0.33 (0.06)	<0.001***
Sensory versus Techniques	0.18 (0.07)	0.116
Communication versus Techniques	−0.15 (0.06)	0.070

*SE*: standard error.

*p < 0.05; ***p < 0.001.

### Diagnosis-type comparisons across categories

Neurodiversity affirming adaptations were rated significantly higher for those with formal diagnoses (*M* = 4.33, *SD* = 0.55) than those with self-identified diagnoses (*M* = 4.09, *SD* = 0.64), *t*(128) = 2.343, *p* = 0.021, *d* = 0.41. There was no statistical difference on any other accommodation category compared (all *p*s > 0.05).

### Additional adaptations

Of the 130 participants, 40 (30.8%) provided open-ended responses yielding 58 codes of individual adaptations they had found helpful. These included repeating five adaptations covered in the questionnaire (providing response options, psychologists being autistic or neurodivergent themselves [three participants], explaining terminology in questionnaires, and spending more time to get to know client) and one response not categorized as an adaptation (benefits of diagnosis). Some adaptations were shared by more than one participant (e.g. four participants raised therapist self-disclosure), resulting in a total of 37 additional adaptations participants found helpful in previous therapy. Of these, 22 (59.46%) were consistent with existing categories, and 15 (40.54%) did not clearly fit into existing categories, see Supplementary Table 1.

Participants raised adaptations consistent with existing categories of communication, information, modality, neurodiversity-affirming, sensory, and techniques. Communication included psychologists adapting their own communication style, using self-disclosure, and tailoring definitions. Information included providing support navigation help, session outlines and summaries, and providing psychoeducation about autism and co-occurring conditions. Modality included accepting journal entries via email, providing location/mode options (home visits, walk and talk therapy), and offering website/webchat options. Neurodiversity affirming including not pathologizing autism traits such as, “not highlighting eye contact as an issue” and “seeing being autistic as a positive difference, using affirming language not deficit-based language.” Sensory adaptations suggested included providing flexible seating options, explicit consent to, “be comfortable in the room however I best felt comfortable,” and role-modeling this, and ensuring sensory needs are met first in the therapy process. Techniques included providing alternatives to cognitive behavior therapy, avoiding triggering reaction sensitive dysphoria and helping to work on this, providing non-talking therapies, allowing therapy dog to join sessions, and providing support to unpack feelings as emotional or physiological.

Additional adaptations not fitting existing categories were grouped into new categories of general good practice, financial, practical, structure, and style. General good practice included “Therapist is authentic, genuine, responsive to feedback, and emotionally expressive,” remembering and recalling “important dates/people/items/events,” and listening, accepting and validating. Financial included being affordable, not billing focused, and providing informed consent of costs and benefits of services to “make a supported choice.” Practical adaptations raised included booking sessions personally, rather than by reception staff, coordinating appointments with other providers (rather than asking clients to arrange), structuring appointment bookings so there is no wait in the waiting room, providing a regular appointment time, and sending reminder texts. Structure adaptations included allowing the client to send an agenda and using routine questions to start sessions. Style adaptations included “client-led therapy” and “more informal and friendly.”

## Discussion

We investigated autistic people’s perspectives on adaptations to therapy raised by clinicians. The importance of this topic to autistic adults in the community was underscored by the rapid participation of volunteer participants with over 100 participants opening the survey within the first week of recruitment. We found endorsement at a group level for about half of the adaptations, but with substantial individual differences. Most items in our list of adaptations could be categorized into internally consistent scales with the category of neurodiversity-affirming practices rated most highly, particularly for participants with a formal diagnosis. Participants also shared additional adaptation ideas. Findings are discussed in reference to literature, limitations, future directions, and implications.

Ratings of each adaptation were mixed at a group level. Approximately half (47.27%) of the proposed adaptations were rated as helpful or extremely helpful aligning with recommendations from clinicians in previous research (Cooper et al., 2018; Petty et al., 2021, 2023). However, 45% were rated as neutral to helpful, with four practices rated as below neutral (3) at a group level. This suggests that there may be important differences between what autistic people themselves feel is helpful and what practitioners perceive is helpful. For example, Cooper et al. (2018) included shorter suggestions as a possible adaptation to CBT for autistic clients, but at a group level our participants rated this as unhelpful. We note, however, that Cooper et al. found use of shorter sessions with autistic clients by only 28% of clinicians. It may be that, similarly to our findings where some individual participants rated shorter sessions as very helpful, clinicians in this study found this adaptation useful for only a subset of autistic individuals. Nevertheless, our findings highlight it should not be assumed that shorter sessions would be helpful for all autistic clients. Furthermore, there may be context-dependent differences in adaptations being helpful with previous research relating to specific modalities (in Cooper et al.’s case to CBT), or countries (i.e. UK) and their associated mental health systems.

At an individual level, we found diversity of responses with no adaptations universally endorsed (i.e. no individual item was rated as only helpful or extremely helpful). This is consistent with the diversity of mental health care experiences shared in previous qualitative research by autistic people (Brede et al., 2022). Furthermore, this diversity is consistent with the significant heterogeneity between autistic individuals in terms of core characteristics and associated features (e.g. presence vs absence of alexithymia) that may impact preferences and priorities for therapy. Similarly, diversity of responses from autistic people aligns with previous research with clinicians that has found a mixture of use of recommended adaptations (Cooper et al., 2018) which may reflect individualizing to specific clients or groups of clients. These findings highlight the importance of not assuming a list of potential adaptations are applicable, wanted, or needed by all autistic clients, but rather highlight the importance of understanding each individual client’s preferences and priorities.

We grouped items into categories based on addressing similar themes which yielded five internally consistent subscales of adaptations (communication, information, neurodiversity-affirming, sensory, and techniques). Consistent with increasing calls in the field for neurodiversity-affirming mental health care both in Australia (e.g. see draft National Autism StrategyAustralian Government Department of Social Services, 2024) and internationally (Chapman & Botha, 2023; Pantazakos & Vanaken, 2023), this category of adaptations was the highest rated by our participants. This was particularly for participants with formal versus self-identified diagnoses. Of note in interpreting group differences there was not a significant difference between clinical characteristics as measured on the AQ-10, suggesting the magnitude of preference is not likely due to differences in levels of characteristics. Instead, it is postulated that greater preferences may reflect individuals with a clinical diagnosis potentially having better self-knowledge, more experience with self-advocating in therapy, less internalized stigma around autism, and/or less propensity toward masking behaviors. We did not explore general knowledge of participants themselves about autism or what it means for them to be autistic, nor strengths and difficulties associated with autism, which may impact preferred adaptations. Future research is needed to explore these possibilities.

The next most helpfully rated category was information (e.g. providing information in advance about session content). Provision of information being helpful may align with autistic thinking styles and a preference for routine and structure through providing this for clients. In addition, information may mitigate uncertainty, given intolerance of uncertainty has been implicated in elevated anxiety rates in autistic people (Boulter et al., 2014; Jenkinson et al., 2020).

The third most helpful category was sensory adaptations including providing options to adjust the environment to sensory needs as well as providing low sensory environments. The finding that overall these were rated as helpful is consistent with sensory processing differences being a core characteristic experienced by autistic people, and aligns with previous qualitative research highlighting the substantial challenges raised by the sensory environment of typical mental health environment service settings (Brede et al., 2022).

Finally, communication and techniques were rated lowest and similarly to each other. While for some individuals, adaptations in these areas were rated as highly helpful, at a group level, these were rated lower. It may be that our sample who were able to complete a written online survey, and highly educated, may be less likely to require communication adaptations. Finally, our list of techniques was somewhat limited and exploration of specific therapies for example may yield additional information as indicated by additional adaptations described by participants in open-ended responses. It is noted that our focus was more on adapting existing therapies rather than comparing preferences for a specific therapy modality per se.

### Additional adaptations

Asking autistic people about their experiences yielded additional ideas for adaptations to therapy which included ideas fitting with our proposed scales (communication, information, modality, neurodiversity-affirming, sensory, and techniques) as well as items suggesting a potential need for additional scales/areas (general good practice, financial, practical, structure, and style). It was observed that suggestions included practices with mixed empirical research to date. For example, practices with a need for further high-quality research such as the use of therapy dogs (see review of animal assisted interventions, Nieforth et al., 2023), through to antecedent-based strategies (e.g. adjusting the sensory environment) classified as evidence-based (Steinbrenner et al., 2020) were shared. Suggestions thus highlight important potential areas for further research and are important to incorporate in updating our tool to expand on existing items and scales and to include these important suggestions from autistic people.

### Limitations and future research

Results should be interpreted in line with the following limitations. First, our sample were predominantly female and highly educated, with self-identified literacy skills to complete an online survey and had been able to access mental health supports in the past. Furthermore, our team all identified as female, inclusion of male researchers, non-speaking individuals, and more inclusive methodologies (e.g. interviews, photographs, or response audio recording) could support participation of a broader more representative sample of the autistic population. Second, we included a double-barreled item of including family members or partners in therapy; in future research separating these may yield differing results for each supporter category. Third, we explored autistic client perspectives, autistic and non-autistic clinician research could yield further valuable insights in terms of legal and practical barriers and enablers to implementation of adaptations rated by autistic people. Exploring why clients and clinicians find specific adaptations helpful could provide further insights. Fourth, we used a scale developed for the purpose of this study. Our main aim was to explore the individual items with a grouping of items for exploration. While initial psychometrics (face and content validity and reliability) show promise, our aim was not to create a scale per se; expanding this work through including additional adaptations raised by participants would be of value to further scale development including external validation by both clinicians and autistic people. Fifth, we note we defined neurodiversity-affirming therapy to include embracing differences in brains and providing supports to affirm neurodivergent identity, however, definitions of neurodiversity in the literature and practice vary (Chapman, 2020). Future research operationalizing neurodiversity-affirming therapy codesigned with autistic people is needed. Finally, we note there is a need for further research into how best to evaluate and assess therapy adaptations for autistic people and to incorporate this in practice. For example, evaluating use of our tool in clinical and therapy decision-making. At a broader level we acknowledge adaptations of existing supports are one, but not the only step required. Participants in our study, as well as research more broadly, have highlighted the need for research into autism-specific mechanisms and formulations for mental health conditions (e.g. intolerance of uncertainty, Boulter et al., 2014; Conner et al., 2022; Jenkinson et al., 2020), and therapies and measurement tools designed with, and for, autistic people (Jones, 2022).

### Implications

Our findings indicate practitioners need to both understand autism broadly, and to actively seek their autistic clients’ input into ways to adapt therapy to their specific strengths and needs. For example, practitioners could ask clients their preferred modality and location (e.g. online, synchronous/asynchronous, and incorporating walking/talking), communication preferences (e.g. directiveness, emotional expressiveness, and ways for them and their clients to provide information before, during, and after sessions) and preferred terminology to describe autism; complete training in autism and neurodiversity-affirming practice; and conduct an environment audit of their clinic room and associated spaces to consider potential adaptations to the sensory environment they could make available (e.g. dimmer switches, curtains, or options to change music). In this way clinicians could improve their knowledge, available skills, and practical resources to facilitate individualization for autistic clients. To support these efforts, adaptations outlined in this measure we have collated into a tool for practitioners to share with clients to identify and inform potential practice (available at https://osf.io/hq3x5). Practitioners could consider which adaptations they could make available (e.g. it may not be possible to install different overhead lighting but to offer alternative accommodations such as lamps) and use such a list to invite client feedback prior to therapy to adapt accordingly, and to use this as a check-in during therapy as preferences, strengths, and needs may change over time. Finally, practitioners are encouraged to consider the content of treatment materials, assessment measures, formulation models and proposed mechanisms of change in the context of autism-related characteristics (e.g. social communication differences), strengths (e.g. details-focused cognitive style), and co-occurring features (e.g. alexithymia).

## Conclusion

Our research addressed an important topic of how to make therapy better for autistic people through evaluating autistic perspectives on adaptations to therapy previously recommended by clinicians. Our findings highlight the importance at an overall level of incorporating neurodiversity-affirming adaptations into practice as well as underscoring the need to understand the individual client with no universally endorsed adaptations. We hope in future, such work will help to bridge the significant mental health needs of autistic people and support better outcomes.

## Supplemental Material

sj-docx-1-aut-10.1177_13623613251313569 – Supplemental material for How can we make therapy better for autistic adults? Autistic adults’ ratings of helpfulness of adaptations to therapy Supplemental material, sj-docx-1-aut-10.1177_13623613251313569 for How can we make therapy better for autistic adults? Autistic adults’ ratings of helpfulness of adaptations to therapy by Jessica Paynter, Kristyn Sommer and Amanda Cook in Autism

## Notes

1. We acknowledge the diversity in preferences in how we describe autism and have elected to use identity-first language (i.e. autistic adults) in line with findings of recent Australian research (Bury et al., 2023).
