# Supplementary material for: How can we make therapy better for autistic adults? Autistic adults’ ratings of helpfulness of adaptations to therapy
Source: Autism. 2025 Jan 22;29(6):1540–53. doi: 10.1177/13623613251313569 (PMC12089669; doi:10.1177/13623613251313569)
Supplement: sj-docx-1-aut-10.1177_13623613251313569 – Supplemental material for How can we make therapy better for autistic adults? Autistic adults’ ratings of helpfulness of adaptations to therapy [file sj-docx-1-aut-10.1177_13623613251313569.docx]

**Supplementary Table 1**

*Participant-reported Helpful Adaptations to Previous Therapy*

| **Category** | **Code** | **n** | **Representative Quotes** |
| --- | --- | --- | --- |
| ***Existing Categories*** |  |  |  |
| **Communication** | Adapt communication style | 5 | *“Speaking slowly, seeking clarification”* |
|  |  |  | *“Psychologist was willing to 'debate' e.g. used a logical framework to help me better understand potential maladaptive thoughts”* |
|  | Self-disclosure | 4 | *“...disclosing that they are also ADHD/ queer / have feminist values”* |
|  |  |  | *“...sharing some of their life to illustrate points”* |
|  | Tailoring definitions | 1 | *“Tailoring definitions and concepts to my personal circumstances (e.g. explaining practicing mindfulness like training for a sport).”* |
| **Information** | Support Navigation help | 3 | *“...someone who helps find the right psychologist or psychiatrist for ourselves.”* |
|  |  |  | *“Advice on who can give diagnoses, etc.”* |
|  | Provide session outlines and summaries | 3 | *“Summary of the therapy emailed to me afterwards”* |
|  |  |  | *“Providing outline of session”* |
|  | Psychoeducation | 1 | *“…discussion of how autism can interact with other conditions like anxiety.”* |
| **Modality** | Open to receiving journal entries via email | 1 | *“…psychologist open to receiving journal entries via email”* |
|  | Location/mode | 2 | *“Home visits”* |
|  |  |  | *“…walk for the first half of our session which was really good...was easier for me to talk not sitting and facing her and moving my body at the same time.”* |
|  | Website/chat options | 1 | *“Website preferably with chat options”* |
| **Neurodiversity Affirming** | Not pathologising autism traits | 2 | *“Not highlighting difficulties with eye contact as an issue”* |
|  |  |  | *“Seeing being autistic as a positive difference, using affirming language not deficit-based language but recognising that being neurodivergent comes with struggles based on neurotypical expectations.”* |

| **Sensory** | Explicit consent and role modelling sensory adaptations | 1 | *“…state that I could be comfortable in the room however I best felt comfortable. She followed that with providing an example of what/how she likes to be comfortable within the room (e.g. a cardigan draped over her lap)....This was helpful as I was able to move/rearrange the cushions and move/adjust myself as needed/wanted.”* |
| --- | --- | --- | --- |
|  | Providing flexible seating options | 1 | *“The ability to choose seating options (i.e. different chairs of different fabrics), the ability to sit on the floor or rug, different pillows that could be held”* |
|  | Sensory needs met first in therapy process | 1 | *“Not do trauma treatment until sensory needs are met in daily life”* |
| **Techniques** | Alternatives to CBT | 1 | *“I don't just need to CBT my brain into thinking positively...I need other strategies to manage them.”* |
|  | Avoid triggering RSD/help improve it | 1 | *“Strong emphasis on avoiding triggering RSD [Reaction Sensitive Dysphoria] and help to improve it.”* |
|  | Non-talking therapies | 1 | *“Non-talking therapies. I cannot remember the name, but two vibrating controls were held, one in each hand and they alternated their vibing. For myself, it triggered long forgotten memories.”* |
|  | Therapy dog | 1 | *“Being allowed to bring my therapy companion dog along,”* |
|  | Unpack feelings as emotional or physiological | 1 | *“When we backtrack to figure out if 'feelings' are to do with emotion or physiological sensation, or analyse both at the same time when trying to process something”* |
| ***New Categories*** |  |  |  |
| **General good practice** | Authentic, genuine, responsive to feedback | 1 | *Therapist is authentic, genuine, responsive to feedback, and emotionally expressive* |
|  | Listening/ Accepting / Validating | 3 | *“Accessible and accepting. listens to patient…”* |
|  |  |  | *“Just being accepting of me as I am.”* |
|  | Remembering key information | 1 | *When my therapist remembers and recalls important dates/ people/ items/ events without prompting* |

| **Financial** | Affordable | 1 | *“….not going to cost an arm and a leg. And assessable for those not on NDIS yet…”* |
| --- | --- | --- | --- |
|  | Not billing focussed | 1 | *“…not being focused on billing - as a NDIS client, I feel like a cash cow”* |
|  | Informed consent about costs | 1 | *“…when asked about formalising diagnosis the process costs (financial emotional…)…were discussed.”* |
| **Practical** | Book with client personally | 1 | *“…my psychologist would book my next session with me personally instead of sending me to the receptionist to book next* |
|  | Coordinates for client | 1 | *Coordinates contact/appointments with other providers instead of just giving information and telling patient to do this themselves.* |
|  | No wait in waiting room | 1 | *No wait in the waiting room - my psych often has a small gap between sessions on purpose, so if you arrive on time you always walk straight in to the session without waiting for another session that may have run overtime.* |
|  | Reminder texts | 2 | *“Reminder texts”* |
|  | Regular appointment time | 1 | *“Regular appointment time put aside for you so that your appointment times don't change each time.”* |
| **Structure** | Client sends agenda | 2 | *“My psychologist being supportive of me sending an agenda for the session”* |
|  | Routine questions to start sessions | 1 | *“Having a routine list of questions to start the session.”* |
| **Style** | Client-led | 2 | *“Client led therapy”*  *“being allowed to go on roundabout 'rants' (usually about work) that describe how/why I'm feeling”* |
|  | More informal and friendly | 1 | *...more informal and friendly* |

*n =* number of codes; quotes edited for minor typographical errors e.g., untill to until.
